# Infants Learn to Follow Gaze in Stages: Evidence Confirming a Robotic Prediction

**DOI:** 10.1162/opmi_a_00049

**Published:** 2021-11-25

**Authors:** Priya Silverstein, Jinzhi Feng, Gert Westermann, Eugenio Parise, Katherine E. Twomey

**Affiliations:** Psychology Department, Lancaster University, UK; Institute for Globally Distributed Open Research and Education; Psychology Department, Lancaster University, UK; School of Psychology and Neuroscience, University of St Andrews, UK; Psychology Department, Lancaster University, UK; Psychology Department, Lancaster University, UK; CIMeC, Center for Mind/Brain Sciences, University of Trento, Italy; Division of Human Communication, Development and Hearing, University of Manchester, UK

**Keywords:** cognitive development, developmental robotics, gaze following

## Abstract

Gaze following is an early-emerging skill in infancy argued to be fundamental to joint attention and later language development. However, how gaze following emerges is a topic of great debate. Representational theories assume that in order to follow adults’ gaze, infants must have a rich sensitivity to adults’ communicative intention from birth. In contrast, learning-based theories hold that infants may learn to gaze follow based on low-level social reinforcement, without the need to understand others’ mental states. Nagai et al. (2006) successfully taught a robot to gaze follow through social reinforcement and found that the robot learned in stages: first in the horizontal plane, and later in the vertical plane—a prediction that does not follow from representational theories. In the current study, we tested this prediction in an eye-tracking paradigm. Six-month-olds did not follow gaze in either the horizontal or vertical plane, whereas 12-month-olds and 18-month-olds only followed gaze in the horizontal plane. These results confirm the core prediction of the robot model, suggesting that children may also learn to gaze follow through social reinforcement coupled with a structured learning environment.

## INTRODUCTION

Gaze following, or the ability to look where a social partner is looking, is a critical milestone in human sociocognitive development (Tomasello, 1995). Although gaze following has been found in many nonhuman species (e.g., Bugnyar et al., 2004; Okamoto-Barth et al., 2007; Povinelli et al., 1996; Tomasello et al., 2007), in human infants this skill has been argued to be a fundamental component of socialization, and infants’ early gaze following has been linked repeatedly to their later language development (e.g., Brooks & Meltzoff, 2005; Carpenter et al., 1998; Morales et al., 2000). Gaze following appears to emerge from around five months of age (Gredebäck et al., 2018; Senju & Csibra, 2008; Szufnarowska et al., 2014) including in non-WEIRD (Western, educated, industrialized, rich, and democratic) populations (Hernik & Broesch, 2018). Thus, in human infants, gaze following appears to be an early-emerging, universal ability, which is intimately linked to our uniquely human social capacities.

While gaze following in infancy has been repeatedly demonstrated, the origins of this ability and the cognitive representations at play remain controversial. Some influential representational theories assume that gaze following relies on infants’ capacity (not learned and present from birth) to be sensitive to others’ communicative intentions or mental states (e.g., Baron-Cohen, 1995; Csibra & Gergely, 2009). On these accounts, infants are born with a receptivity to adults as intentional communicative agents, and based on this, look where adults look in order to obtain information. In support of these accounts are studies that suggest infants may have some rudimentary form of gaze following (gaze cueing) from birth, evidenced by the finding that newborns detect an object on a screen faster if it appears in a location previously cued by another’s gaze (Farroni et al., 2004). Others argue that although this is not an ability that is present from birth, at a certain time point the ability to read others’ intentions and hence follow gaze “switches on,” supported by the finding that 6-month-old infants need the presence of ostensive cues (direct gaze and infant directed speech) in order to follow gaze (Hernik & Broesch, 2018; Senju & Csibra, 2008).

However, whether infants’ ability to follow gaze is based on their existing understanding that adults are intentional communicative agents has been subject to debate. For example, gaze cueing in newborns is not robust and is found only when the motion of the schematic gaze shift is visible (Farroni et al., 2004), leaving open the possibility that apparent gaze following reflects merely a sensitivity to motion cues. Similarly, although 6-month-olds follow gaze in the presence of ostensive cues (Senju & Csibra, 2008), this has also been found for the non-communicative action of shivering (Gredebäck et al., 2018; Szufnarowska et al., 2014) and in a larger study (with the power to detect smaller effects), even without any cue at all (Gredebäck et al., 2018), suggesting that infants may simply be more likely to gaze follow even in noncommunicative situations in which their attention has been captured. Similarly, although the finding that 5- to 7-month-old infants in Vanuatu, a culture with fewer face-to-face parent-infant interactions, show gaze following (Hernik & Broesch, 2018) can be used to argue for a capacity for this ability that is present from birth, this is cautioned against by the authors themselves, and gaze following in this paradigm (originally used by Senju & Csibra, 2008) could also be explained by motion tracking, or following of the face contour (von Hofsten et al., 2005). These and similar results highlight the importance of designing paradigms that assess gaze following absent of these confounds.

In contrast to these representational accounts, learning-based accounts of gaze following assume that infants learn this skill via reinforcement learning. Specifically, during interaction, a caregiver may avert their gaze to look at something interesting entering the environment. If the infant by chance looks in the same direction, they are rewarded by the interesting information, and will be more likely to look in the same direction as the caregiver in the future (e.g., Triesch et al., 2006). In this scenario, social scaffolding by the caregiver, for example, holding objects directly in the infant’s field of view and rapid alternation of head turns from infant to object, may increase opportunities for learning (Moore & Corkum, 1994). Alternatively, infants may learn to follow gaze by initially learning that the caregiver’s hands are a reliable cue to the focus of the caregiver’s attention (Deák et al., 2014; Yu & Smith, 2013). Although the mechanisms proposed in learning-based theories differ, critically, all share the assumption that, at least early in development, associative learning and reinforcement are sufficient to support the emergence of gaze following given a suitably structured learning environment—without the need for any specific, preexisting sensitivities to communicative or referential intent.

In the current article we focus on the predictions of a developmental robotic simulation of the emergence of gaze following based on low-level learning mechanisms. Nagai et al. (2006) successfully taught a developmental robot with a simple neural network cognitive architecture to gaze follow through supervised associative learning. The robot was equipped with a camera that fed images of a human experimenter to a visual system consisting of an artificial neural network map, which encoded these images, and a retinal smoothing layer, simulating the development of infants’ visual acuity. Training consisted of five steps. First, the robot’s camera was focused on the experimenter’s face. Next, the experimenter held up an object and shifted her gaze toward it. Visual input from the camera was then processed by the neural network after smoothing, generating a representation of the experimenter’s face. Based on this representation, the robot generated a motor command, adjusting the joint angles in its head and neck, resulting in a head turn and a change in its visual field. The robot was then given feedback based on the output error between the location of the object in the visual field and its gaze direction: if the object was centered in the visual field, gaze following was considered successful and no adjustments to the neural network were made. When the object was outside the visual field or off-center, random noise was added to the connection weights in the robot’s neural network. Across training, therefore, head movements resulting in incorrect gaze following were less likely to be produced, increasing the relative strength of connections which produced correct gaze following.^1^ Following training, testing with previously untrained images demonstrated that the robot learned to follow the experimenter’s gaze. Thus, this work raises the possibility that low-level perceptual and proprioceptive information coupled with social reinforcement are sufficient to support the emergence of this important ability.

However, testing during training revealed that in the robot, the ability to follow gaze developed in stages: early in learning, the robot initially learned to follow gaze in the horizontal plane, and only later in the vertical plane. The mechanism underlying this developmental trajectory relates to characteristics of the visual input to the system. Specifically, there was more variability in the images that varied along the horizontal plane, providing a richer input to learning than the less variable vertical plane. Thus, this work makes the empirically testable prediction that, if infants also learn to gaze follow on the basis of similar low-level mechanisms, they too should initially follow gaze shifts more successfully in the horizontal than the vertical direction. Importantly, the same prediction does not follow clearly from representational theories that assume an ability to understand others’ communicative intent, and therefore predict that infants should be able to follow gaze irrespective of gaze direction.

It is important to note that, when evaluating low-level and higher level accounts (also called “lean” and “rich” interpretations; Haith, 1998) of infant cognition against each other, evidence for a low level explanation is usually also compatible with a high-level account (but not vice versa). For example, in the case of gaze following, if infants’ behavior can be explained on the basis of reinforcement and learning, infants may nevertheless also understand intentions. It is therefore not possible to directly falsify high-level accounts by showing that data are compatible with low-level accounts; it is only possible to show that a certain behavior is compatible with low-level accounts, therefore making the assumption of more complex high-level abilities unnecessary. In the current case, we know that the robot model, which exhibited the horizontal-then-vertical gaze following behavior, did not have any understanding of intentionality. To our knowledge, there is no other account making the same prediction, and specifically, it does not seem that any account assuming intention understanding in infants would make this prediction. Critically, an important means to verify the validity of a computational model of developmental processes is to test if its predictions are borne out in studies with children (Asada et al., 2009; Morse & Cangelosi, 2017; Schlesinger & McMurray, 2012; Westermann & Mareschal, 2012). In the present study we therefore provide this critical test. Specifically, we ask whether, like the robot, infants learn to gaze follow in stages, acquiring horizontal gaze following before vertical gaze following. If this is the case, this would confirm a central prediction of the robot model.

A pilot study showed that 12-month-old infants were able to follow the gaze direction in our stimuli and were not at ceiling or floor. This age group are therefore at an intermediate developmental stage in which gaze following is not consistently accurate, raising the possibility that at this age, we may observe the differences in horizontal/vertical tracking predicted by the robot. Therefore, in Experiment 1 participants were 12-month-old infants. In order to investigate the developmental trajectory of gaze following, we then tested 6-month-olds (Experiment 2) and 18-month-olds (Experiment 3). This study was preregistered (https://osf.io/e73fw) and all materials, code, and data can be found in the Supplemental Materials on the Open Science Framework (OSF; https://osf.io/fqp8z/).

## EXPERIMENT 1 (12-MONTH-OLDS)

### Method

#### Ethics.

All data were kept confidential. All three experiments were approved by the university ethics committee and adhered to the British Psychological Society guidelines.

#### Participants.

Participants were recruited from a database of families at Lancaster University Babylab, and were given a book as a gift for participation and £10 travel reimbursement. Although an individual measure of socioeconomic status was not collected, the average income in Lancaster is £24,000 annually, which is slightly less than the national average (£29,000). Infants were not recruited for the study if they had any developmental delays or visual impairments. Parents gave informed, written consent before participation, and were free to withdraw their consent. Sample size was determined using Bayesian sequential testing (Schönbrodt et al., 2017). In Bayesian sequential testing, Bayes factors are calculated after every participant (pending a predefined minimum number of participants) until a preregistered Bayes factor threshold is reached for all analyses run (or a predefined maximum number of participants is reached). We determined a minimum a priori sample size of 20 infants, with a maximum of 40 infants, and set a threshold of a BF_10_ (i.e., evidence for the research hypothesis) and BF_01_ (i.e., evidence for the null) of 10 or above for all preregistered analyses. We did not reach this threshold for all tests when we reached our maximum number of infants. Therefore, our final sample consisted of 40 typically developing 12-month-old infants (mean age: 364 days; range: 349 days to 383 days; 27 female; all Caucasian; 34 monolingual English). Infants were excluded if they provided less than 50% of usable trials in which they were not crying (*N* = 4).

#### Stimuli and Design.

**Figure 1.**
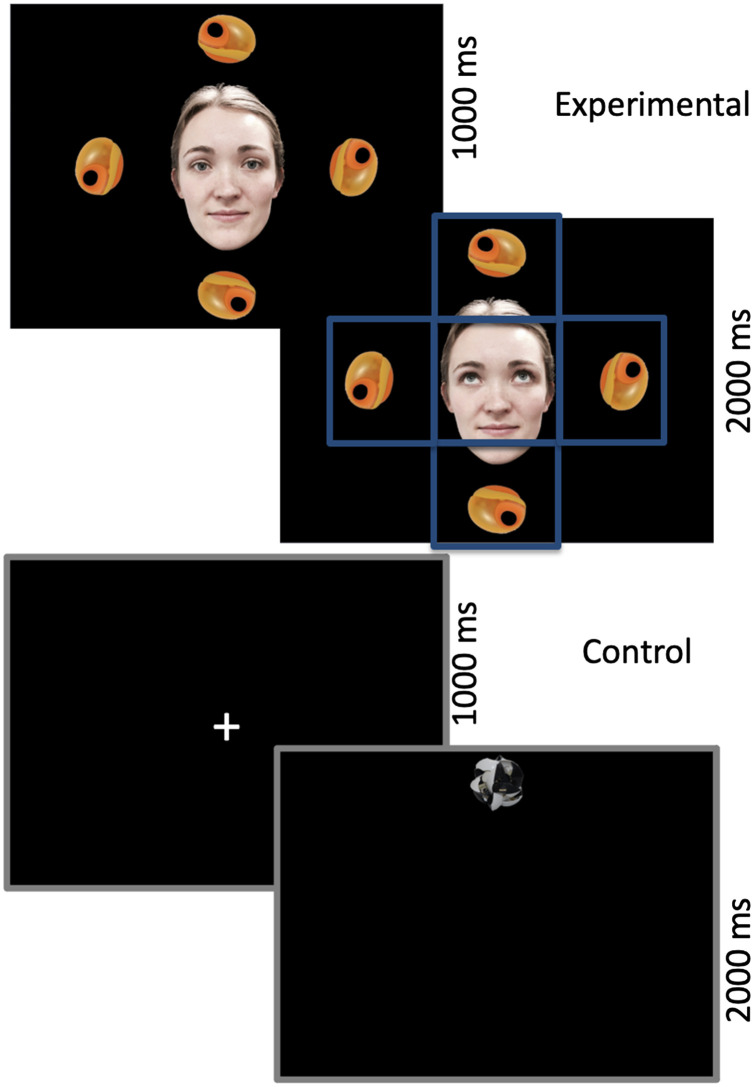
**Experimental and control conditions.** Central cue (direct gaze or fixation cross) for 1,000 ms, followed by a directional cue (averted gaze or object) for 2,000 ms. Blue boxes indicate areas of interest (AOIs) (visual angle of center AOI = 9.57° × 9.57°; visual angle of object AOI = 9.57° × 8.89°).

Example stimuli are depicted in Figure 1. To construct our stimuli we used five photographs of one female face looking left, right, up, down, and directly at the camera. The eyes from the left, right, up, and down photographs were superimposed onto the face looking directly at the camera, ensuring the face was identical apart from gaze direction. The use of static images (as opposed to videos) has been shown to effectively elicit gaze following in 12-month-olds (von Hofsten et al., 2005). Target objects were selected from the NOUN database (Horst & Hout, 2016) that had no obvious top/bottom in order to avoid biasing infants’ attention.

Trials were split into control trials and experimental trials. Each trial consisted of a 3-s-long video. Control trials consisted of a central fixation cross (1,000 ms) followed by a novel object appearing in one of four locations at 200 pixels left, right, up, or down from the center of the screen (2,000 ms), and were designed to test whether infants found gaze shifts (without gaze following) a priori easier in the horizontal than the vertical plane. Experimental trials consisted of a human face (visual angle: 12.96° × 8.89°) looking directly at the infant, surrounded by four images of the same object in each of the locations (1,000 ms) followed by the same face with averted gaze to one of the four exemplars (2,000 ms). In total, 64 videos were made, consisting of the eight objects (average visual angle = 4.11° × 4.79°) in the four locations for both the control and experimental conditions. These videos were pseudorandomized into four orders such that infants never saw the same object, location, or trial type (control, experimental) on more than two successive trials. Between trials there was a variable animated attention-getter. All videos are available on OSF.

#### Procedure.

Infants sat on their caregiver’s lap during the experiment approximately 0.6 meters away from a 23-in. screen. Parents were instructed to close their eyes during the experiment to ensure they were blind to the stimuli. An eye-tracker (Tobii X120) captured infant looking times and gaze locations on screen. We used Tobii Studio 3.3.1 to present stimuli and gather eye-tracking data. We performed a 5-point calibration for all infants before beginning the experiment. After this calibration, we instructed caregivers not to talk to or interact with their infant, and that they could stop the experiment at any time if the infant became too fussy, and the experiment began. Infants saw up to a maximum of 64 trials in one of the four pseudorandomized orders (range: 28–64, *M* = 57; *SD* = 9).

#### Analysis.

We performed all analyses in R 3.5.2 (R Core Team, 2017). We exported raw data from Tobii Studio 3.3.1 and analyzed them using a combination of the eyetrackingR package (Dink & Ferguson, 2015) and our own code (all code is available on the OSF). Areas of interest were defined for center (400 × 400 pixels), and left, right, up, and down object locations (each 340 × 400 pixels). All preregistered analyses are reported either in the main article or in the Supplemental Materials on OSF. Our outcome variables included two measures of accuracy: proportion looking (length of looking at target AOI divided by total looking at all four object AOIs); dichotomous target looking (whether or not the infant looked at the target AOI for any frames after the gaze shift); and a measure of latency (time taken for the infant to look at the target AOI). Corresponding Bayesian analyses were carried out for all frequentist analyses (Dienes & Mclatchie, 2018; Wagenmakers et al., 2018). All Bayesian analyses used a default Bayes factor with a wide Cauchy distribution (scale of effect = 0.707) and were calculated using the BayesFactor R package (Morey & Rouder, 2015). For all Frequentist analyses, we used a significance threshold of *p* < .05. BF_10_ or BF_01_ of more than 3 are considered moderate evidence, using the system outlined by Jeffreys (1961). Raincloud plots were created adapting code from Allen et al. (2019).

### Results

#### Accuracy.

**Figure 2.**
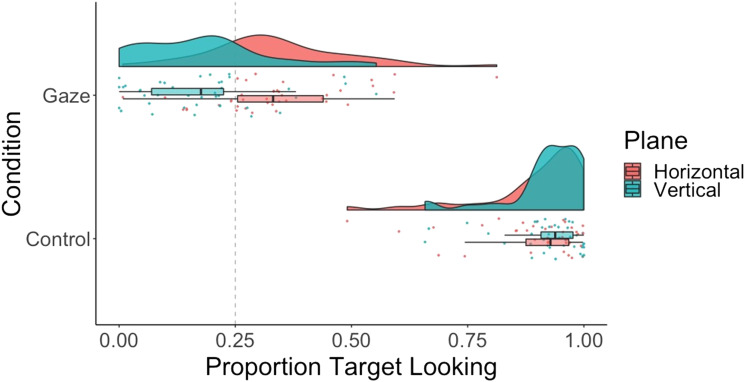
**Raincloud plot showing proportion target looking across conditions (gaze vs. control) and planes (horizontal vs. vertical) for 12-month-olds.** Dashed line indicates chance performance.

**Figure 3.**
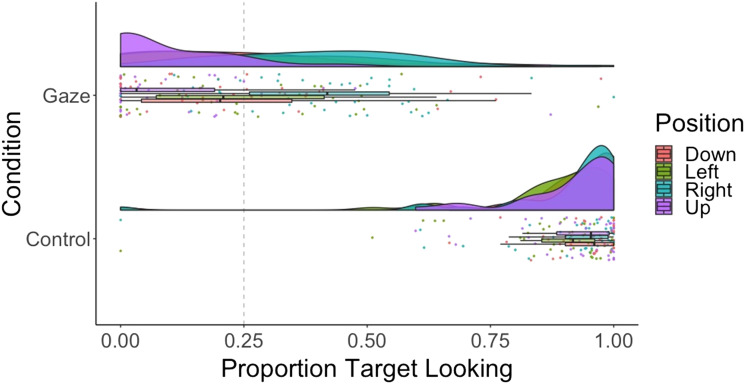
Raincloud plot showing proportion target looking across conditions (gaze vs. control) and positions (down, left, right, up) for 12-month-olds.

We submitted proportion looking to a 2 × 2 repeated measures ANOVA with main effects of condition (gaze vs. control) and plane (horizontal vs. vertical) (Figure 2; see Figure 3 for gaze across individual conditions). Accuracy was higher for control than gaze trials—main effect of condition: *F*(1, 39) = 1,222, *p* < .001; η^2^ = .85; BF_10_ = 2.93e^63^, and higher for the horizontal than the vertical plane—main effect of plane: *F*(1, 39) = 9.02, *p* < .005; η^2^ = .01; BF_10_ = 3.08. The ANOVA revealed a significant interaction between condition and plane, *F*(1, 39) = 23.72, *p* < .001; η^2^ = .02; BF_10_ = 1.65. Planned, two-tailed, paired *t* tests revealed that in the gaze condition, accuracy was higher for the horizontal, *M* = 0.34, *SD* = 0.15, than the vertical plane, *M* = 0.18, *SD* = 0.14; *t*(39) = 4.14, *p* < .001; *d* = 1.09; BF_10_ = 2,774. In contrast, in the control condition, there was no evidence for a difference in accuracy across the two planes—horizontal: *M* = 0.90, *SD* = 0.11; vertical: *M* = 0.92, *SD* = 0.08; *t*(39) = −1.77, *p* = .08; *d* = −.26; BF_01_ = 2.35. Finally, *t* tests against chance (0.5) revealed that in the gaze condition, accuracy was above chance for the horizontal plane, *t*(39) = 3.72, *p* < .001; *d* = .83; BF_10_ = 70, and below chance for the vertical plane, *t*(39) = −3.14, *p* < .01; *d* = −.70; BF_10_ = 15. Dichotomous target looking results mirrored the proportion looking results and can be found in the Supplemental Materials on OSF (Figure S1).

#### Latency.

For trials where infants looked at the target AOI at any point after the gaze shift, we submitted latency to a 2 × 2 repeated measures ANOVA with main effects of condition (gaze vs. control) and plane (horizontal vs. vertical) (Figure S4). The ANOVA revealed moderate evidence for no interaction between condition and plane for latency, *F*(1, 36) = 0.02, *p* = .90; η^2^ = 8.71e^−5^, BF_01_ = 5.66. Latency was shorter for control than gaze trials—main effect of condition: *F*(1, 36) = 20.49, *p* < .001; η^2^ = 1.03e^−1^, BF_10_ = 283—and there was moderate evidence for no difference in latency in the horizontal and vertical planes—main effect of plane: *F*(1, 36) = 0.001, *p* = .98; η^2^ = 6.00e^−6^, BF_01_ = 5.71.

### Discussion

In this experiment we tested the predictions of Nagai et al.’s (2006) robotic implementation of the development of gaze following, specifically that infants in the early stages of gaze following should first learn in the horizontal plane, and then the vertical plane. Our results capture this prediction: they suggest that 12-month-old infants are able to follow horizontal gaze shifts from static images of a face, whereas they are not yet able to do this for vertical gaze shifts. We find no such difference in our control condition, where objects appear in the same locations but are not cued with gaze. We find that latency is faster in the control than the gaze condition, which could be due to it being easier to disengage from the fixation cross than the face, and the lack of competing stimuli to look at. We find no difference in latency across planes, which suggests that infants are not slower to make gaze shifts in either plane. Importantly, the results of our control condition indicate that the difference found in the experimental condition is not because infants have difficulty shifting attention to salient vertical targets. Our results are compatible with learning-based theories, specifically Nagai and colleagues’ robotic implementation, in which a combination of associative learning mechanisms and social reinforcement were sufficient to support the emergence of gaze following. In contrast, representational accounts offer no mechanism by which a difference between horizontal and vertical gaze following might arise. Importantly, however, to strengthen the learning-based account of gaze following, we should be able to demonstrate developmental changes in horizontal and vertical gaze following. Specifically, if gaze following is learned in stages (rather than there being a general bias for horizontal gaze in infants), we should be able to find a stage at which younger infants do not follow either horizontal or vertical gaze. Thus, in Experiment 2, we tested 6-month-old infants in the same paradigm.

Although in Experiment 1, when using Bayesian sequential testing we reached our maximum number of infants, this was due to not meeting our threshold for *all* analyses. This is due to the fact it takes a larger amount of evidence to reach any given Bayes factor in support of the null hypothesis than against it (Johnson & Rossell, 2010). However, our frequentist results were the same at 20 participants as at 40 participants, that is, all results significant at the *p* < .05 threshold at 40 participants also were significant at 20 participants. This is likely due to the high number of trials (64) compared to many infancy studies. Hence, in each further experiment reported, we collected a final sample of 20 infants.

## EXPERIMENT 2 (6-MONTH-OLDS)

### Method

All stimuli and procedures were identical to Experiment 1. Infants viewed up to 64 trials (range: 28–64, *M* = 57; *SD* = 10). As we did not observe a difference between horizontal and vertical gaze following latency in Experiment 1 (12-month-olds), we did not analyze latency in Experiments 2 and 3 (6- and 18-month-olds, respectively).

#### Participants.

Twenty typically developing 6-month-old infants took part (mean age: 182 days; range: 166 days to 196 days; 7 female; all Caucasian; 18 monolingual English). Participants were from the same demographic background as participants in Experiment 1.

### Results

**Figure 4.**
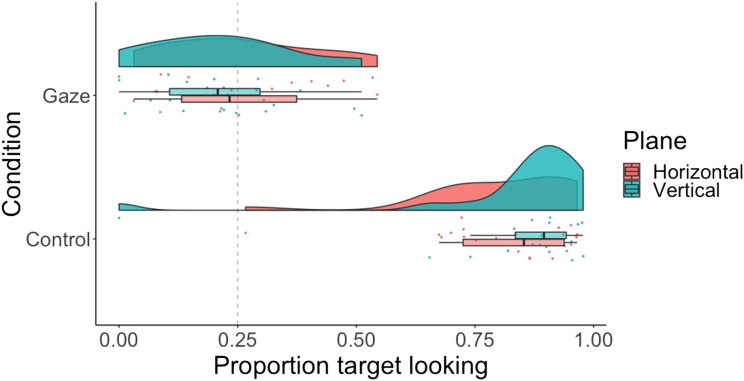
**Raincloud plot showing proportion target looking across conditions (gaze vs. control) and planes (horizontal vs. vertical) for 6-month-olds.** Dashed line indicates chance performance.

We submitted proportion looking to a 2 × 2 repeated measures ANOVA with main effects of condition (gaze vs. control) and plane (horizontal vs. vertical) (Figure 4). Accuracy was significantly higher for control than gaze trials—main effect of condition: *F*(1, 19) = 175, *p* < .001; η^2^ = .75; BF_10_ = 5.09e^21^, and we found moderate evidence for no difference between horizontal and vertical accuracy—main effect of plane: *F*(1, 19) = 0.08, *p* = .78; η^2^ = .0002; BF_01_ = 4.28. The ANOVA revealed moderate evidence for no interaction between condition and plane for proportion looking—horizontal gaze: *M* = 0.33, *SD* = 0.19; vertical gaze: *M* = 0.34, *SD* = 0.23; horizontal control: *M* = 0.91, *SD* = 0.13; vertical control: *M* = 0.87, *SD* = 0.22; *F*(1, 19) = 1.34, *p* = .26; η^2^ = .004; BF_10_ = 3.81. Finally, planned *t* tests found no evidence for accuracy at levels greater than expected by chance for either plane in the gaze condition—horizontal: *t*(19) = 0.36, *p* = .73; *d* = .11; BF_01_ = 3.12; vertical: *t*(19) = −1.13, *p* = .27; *d* = −.36; BF_01_ = 1.96. Dichotomous target looking results mirrored the proportion looking results and can be found in the Supplemental Materials on OSF (Figure S2).

We note that it is possible that for 6-month-olds true effect sizes are smaller, and so we cannot guarantee that a larger sample of infants would not have found effects where we found null results. However, all Bayes factors but one were moderate (BF_01_ of above 3), making this interpretation less likely. The only Bayes factor that was inconclusive is for vertical gaze against chance, which is qualitatively below chance level (as is horizontal gaze against chance).

#### Comparison With Experiment 1.

In order to assess whether 6-month-olds were indeed performing differently to 12-month-olds, we submitted proportion looking in gaze trials to a 2 × 2 mixed ANOVA with main effects of age (12 months vs. 6 months) and plane (horizontal vs. vertical). Although according to our frequentist analysis the ANOVA did not reveal a significant interaction between age and plane for proportion looking, Bayes factor analysis revealed strong evidence for an interaction, *F*(1, 58) = 2.73, *p* = .10; η^2^ = 0.03; BF_10_ = 14.56.

### Discussion

In Experiment 2 we tested whether 6-month-old infants could follow gaze in either the horizontal or vertical plane. In contrast to 12-month-olds, this younger group showed no difference in their horizontal and vertical gaze following and did not show above-chance accuracy in either plane. As in Experiment 1, these results capture the predictions of the robotic model presented by Nagai et al. (2006), suggesting that younger infants have not yet learned to follow gaze in either plane. Thus, the difference in gaze following ability across the horizontal and vertical planes that we find in 12-month-olds is not an artifact of our stimuli (as the stimuli used were identical). Further, infants do not exhibit a general bias to follow only horizontal gaze, strengthening our interpretation that infants may learn to follow gaze in stages.

In Experiment 3, we investigated whether, as we predicted according to the robot model, older infants can follow gaze equally successfully in the horizontal and vertical plane.

## EXPERIMENT 3 (18-MONTH-OLDS)

### Method

All stimuli, procedures, and analyses were identical to Experiments 1 and 2. Infants viewed up to 64 trials (range: 42–64, *M* = 60; *SD* = 7).

#### Participants.

Twenty typically developing 18-month-old infants took part (mean age: 546 days; range: 530 days to 567 days; 10 females; all Caucasian; all monolingual English). Participants were from the same demographic background as participants in Experiment 1. Two additional infants were excluded due to fussiness.

### Results

**Figure 5.**
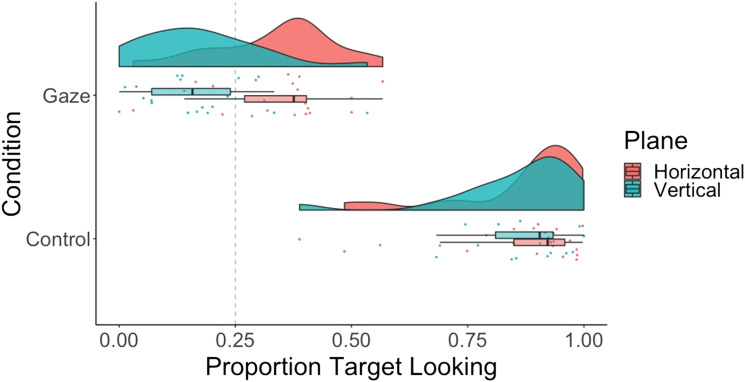
**Raincloud plot showing proportion target looking across conditions (gaze vs. control) and planes (horizontal vs. vertical) for 18-month-olds.** Dashed line indicates chance performance.

**Figure 6.**
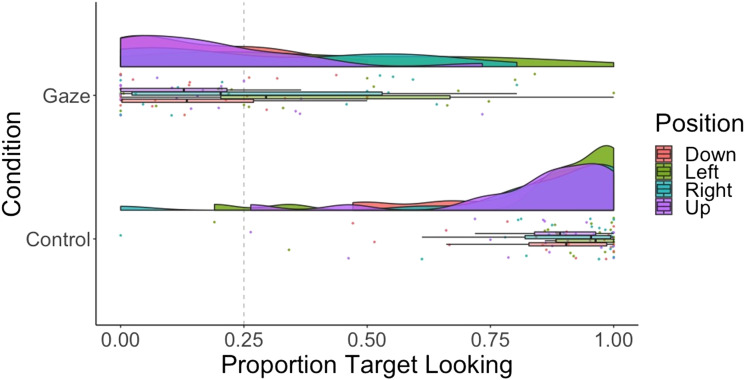
Raincloud plot showing proportion target looking across conditions (gaze vs. control) and positions (down, left, right, up) for 18-month-olds.

We submitted proportion looking to a 2 × 2 repeated measures ANOVA with main effects of condition (gaze vs. control) and plane (horizontal vs. vertical) (Figure 5; see Figure 6 for gaze across individual conditions). Accuracy was greater for control than gaze trials—main effect of condition: *F*(1, 19) = 356, *p* < .001; η^2^ = .82; BF_10_ = 7.38e^26^—and greater for the horizontal than the vertical plane—main effect of plane: *F*(1, 19) = 9.37, *p* < .006; η^2^ = .02; BF_10_ = 2.40. The ANOVA revealed a significant interaction between condition and plane for proportion looking, *F*(1, 19) = 9.92, *p* < .01; η^2^ = .01; BF_10_ = 2.83. Planned, two-tailed, paired *t* tests revealed that in the gaze condition, accuracy was higher for the horizontal, *M* = 0.34, *SD* = 0.13, than the vertical plane, *M* = 0.17, *SD* = 0.13; *t*(19) = 3.52, *p* < .005; *d* = 1.24; BF_10_ = 72.68, with moderate evidence for no difference in the control condition—horizontal: *M* = 0.87, *SD* = 0.14; vertical: *M* = 0.86, *SD* = 0.14; *t*(19) = 0.53, *p* = .60; *d* = .10; BF_01_ = 3.13. Finally, *t* tests against chance (0.5) revealed that in the gaze condition, accuracy was significantly above chance for the horizontal plane, *t*(19) = 2.93, *p* < .01; *d* = .93; BF_10_ = 7.66, and significantly below chance for the vertical plane, *t*(19) = −2.61, *p* < 0.05; *d* = −.82; BF_10_ = 4.07. Dichotomous target looking results mirrored the proportion looking results and can be found on OSF (Figure S3).

### Discussion

In this experiment, we tested whether 18-month-infants were yet able to follow gaze in both the horizontal and vertical plane. We found that they showed the same pattern as the 12-month-olds and were only able to follow gaze in the horizontal plane. Thus, in Experiment 3 we did not identify the age at which infants have acquired both horizontal and vertical gaze following abilities. Critically, however, we replicated the 12-month-old results with 18-month-olds; the results of Experiment 3 are therefore consistent with an account of gaze following in which infants learn in stages, suggesting that infants remain at the horizontal stage in this age group. Note that in all three experiments we investigate gaze following of just the eye gaze shift, which is a much more subtle cue than the more commonly used head turn and eye gaze shift (Moore et al., 1997). Interestingly, we found that rather than being at chance, infants were significantly below chance in their vertical gaze following. Tentatively, we suggest that this could be due to infants persevering with horizontal saccades after successfully following gaze in the horizontal condition, but future research would be needed to determine whether this unexpected effect could even be replicated, and if so, what the mechanism may be.

## GENERAL DISCUSSION

In the current study we tested the predictions of Nagai et al.’s (2006) robotic implementation of the development of gaze following. In Nagai et al. (2006), the robot learned to gaze follow based on associative learning and visuomotor input and did so in stages: first in the horizontal plane, followed by the vertical plane. Our results capture this prediction: while 6-month-old infants are unable to gaze follow in either plane in our paradigm, by 12 months they are able to follow gaze in the horizontal plane, but critically, not the vertical plane. This pattern is, unexpectedly, replicated in 18-month-olds, suggesting that the ability to follow vertical gaze shifts without a head turn may develop after this age. We find no such difference in our control conditions, where objects are not cued with gaze; thus, the difference we observe in the experimental conditions is specific to gaze following and is not driven by motoric differences in the ease of horizontal and vertical eye movements. Our results are therefore compatible with the robotic prediction and learning-based theories of gaze following, suggesting that a combination of associative learning mechanisms and social reinforcement may be sufficient for the emergence of gaze following. Importantly, Nagai and colleagues’ model offers an explicit mechanism by which the horizontal-first gaze following behavior we observed may emerge. In the robotic model, visual input in the horizontal plane was more variable than visual input in the vertical plane. The model therefore used this additional information for learning in the first instance, leading to better gaze following in the horizontal plane early in learning. Thus, taken together with our replication of the model’s behavioral prediction, this work makes the further, empirically testable prediction that gaze input to infants, in particular gaze direction information, should be more variable in the horizontal than the vertical plane.

While the current data offer support for learning-based accounts of gaze following, we cannot rule out alternative accounts based on cognitive processes present from birth. In particular, the shape of the human eye is such that horizontal eye movements are easier to see, due to the amount of visible white sclera being larger and therefore there being a clearer contrast between iris and sclera. For this reason, one could argue that the perceptual system is simply tuned to spot more perceptually contrasting movements (and for evidence of gaze cueing in newborns, see Farroni et al., 2004). However, if this were driving our results, in the current study we might expect to find better gaze following for upward vertical gaze (where there is more visible sclera) than the downward vertical gaze (where no sclera is visible), which is not the case. In fact, we find worse accuracy for upward looks than all three other positions (Figures 3 and 6), in contrast to an upward bias found in adults (Bock et al., 2008).

Intuitively, this is consistent with learning theories, as infants are typically situated below adults’ line of gaze, which would elicit more frequent downwards than upwards looks from the adult. In the same vein, it is also possible that as well as being more perceptually variable, horizontal gaze is just more common in input to infants. It is also important to note that infants can only see an adult’s gaze cues when the adult is facing the infant. In this context, adults may be more likely to exhibit lateral gaze behavior (to the right or left of the infant) as opposed to looking above (i.e., behind) the infant as they engage with them. Gaze behavior while engaging with the infant is also likely to be followed by the adult labeling an object or the infant turning to look in the direction of the adult’s gaze. As a result, this adult gaze bias along the horizontal plane, coupled with feedback from the environment about the lateral object to which the adult is looking, could serve as an associative mechanism for the horizontal gaze bias. Again, empirical evidence from naturalistic input to infants can assess these possibilities.

It is also possible that other morphological features of human eyes or face (e.g. the fact that eyes are arranged in the horizontal axis themselves) are responsible for this effect in human infants, however this would likely affect all eye movements, and so induce a bias in our control condition (which it does not).

Nonetheless, while elements of the learning environment (such as increased variability in the horizontal plane) may shape infants’ behavioral responses, they may still possess an ability to read others’ mental states. However, theories proposing that infants learn to follow gaze by understanding the communicative intentions of others do not offer a mechanism that could explain our observation that infants learn to follow gaze earlier in the horizontal than the vertical plane. For example, as our stimuli begin with an ostensive cue (direct gaze), Natural Pedagogy theory (Csibra & Gergely, 2009, 2011) would predict instead that even the 6-month-olds in Experiment 2 would follow gaze, due to the referential expectation induced by the ostensive cue. We cannot rule out the possibility that some other, representation-based mechanism biases gaze following to horizontal planes. However, we are not aware of any representational theory making this prediction.

Importantly, we know that a learning theory without intention understanding is sufficient to produce this behavior (Nagai et al., 2006), and we interpret our results within a theory that is as simple as possible, and as complex as necessary. We may, however, consider what evidence would be needed to be able to provide definitive support for a capacity present from birth to understand others’ intentions. The strongest evidence would come from evidence of newborn gaze following, which to date has not been found. Newborn gaze *cueing* has been observed (Farroni et al., 2004), but this is not the same as gaze following, and can be explained by a much simpler mechanism. In these studies, a gaze shift is followed by a target appearing on a cued or uncued side. Infants are found to be faster to detect targets that appear on the cued side, which is taken by some to be evidence of rudimentary gaze following ability. However, in these studies infants could be tracking the motion of the gaze shift, and are simply quicker to fixate on a cued target because it is a shorter distance from the cued target than the uncued target to the location of the termination of the movement. Beyond this, it is very difficult (if not impossible) to measure intention understanding itself without a verbal response.

Our paradigm could be altered (by accessing our open materials on the OSF) in many ways to gain further theoretical understanding of what this horizontal bias in infant gaze following means. Although our chosen control condition controls well for any horizontal bias in infant eye movements, other theoretically interesting comparisons could be made to our experimental condition. Using a control that was non-social but still directed infant attention to one of four possible objects would reveal whether infants find it more difficult to disengage from the face vertically than horizontally, independent of social cues. One way to test this would be to have a central face with direct gaze remaining on screen, while one of the four targets flashed.

In addition, our stimuli (by design) were artificial, and so have many features that make them unlike naturalistic social interactions. The face was artificially static and presented alone without a body, the gaze shift was engineered from two pictures as opposed to being a video, the scene was absent of natural clutter, and trials were repetitive. However, we do not believe that the repetitive nature of the paradigm affected our results, as we do not see behavior changing in a systematic way across trials (see Figures S5–S7). Fine-grained control of the stimuli enabled us to effectively investigate the difference between horizontal and vertical gaze following while ensuring horizontal and vertical stimuli were as visually similar as possible. However, future research should investigate whether our findings can be replicated in more naturalistic scenarios.

It is interesting to note that infants in our study are ‘bad’ at gaze following; that is, even the 18-month-olds were only above chance for horizontal gaze following. This is to be expected, as it has been shown repeatedly that infants respond best to head and eye movements in combination (Michel et al., 2021; Moore et al., 1997). The movement of just the eyes is a very subtle cue and so makes it understandably more difficult for infants to disengage from the face, where infants are known to fixate for the majority of the time even when there is a combined head and gaze shift (e.g., von Hofsten et al., 2005). We chose to manipulate only eyes in this experiment in order to create stimuli that were as controlled as possible, and to address a possible confound of combined head and gaze cue stimuli. Studies where head and gaze cues are confounded leave open the possibility that infants may just be attracted to the face contour itself, and so look in the direction that the head is turned, leading to the object in their peripheral vision being closer and more likely to catch their attention (von Hofsten et al., 2005). Nevertheless, future research should explore whether our findings can be replicated for combined head movement and eye cues, since, if gaze following is learned, these additional cues should also be integrated by the learning mechanism.

Finally, it is possible that factors affecting gaze following also affect infant perception of other object related actions, for example, object tracking. Indeed, it has been found that 5- to 9-month-old infants are better at object tracking in the horizontal than vertical plane (Grönqvist et al., 2006). Further research could determine whether these biases are due to a common underlying factor (for example, visual input of a wide variety of scenes being more variable in the horizontal plane, not just gaze events), or whether these are unrelated phenomena.

## CONCLUSION

Taken together, our results and those of Nagai et al. provide evidence that a low-level learning mechanism provided with structured input may be sufficient to support the development of gaze following in human infants. Importantly, however, current prominent theories in developmental psychology (e.g., Baron-Cohen, 1995; Csibra & Gergely, 2009) do not predict that there would be differences in accuracy of gaze following across the horizontal and vertical planes, as an understanding of shared attention or communicative intentions (the basis for these theories) is not dependent on direction of gaze. As such, the current results point to new opportunities for the development of mechanistic and mentalistic theories that can account for these behavioral data.

## ACKNOWLEDGMENTS

We thank Anna Barnett for appearing in the stimuli, Malcolm Wong for help with data collection, Katharina Kaduk for her role as lab manager at Lancaster Babylab, and the infants and caregivers who took part in the study.

## FUNDING INFORMATION

PS, Leverhulme Trust (https://dx.doi.org/10.13039/501100000275), Award ID: DS-2014-014. GW, EP, Economic and Social Research Council (https://dx.doi.org/10.13039/501100000269), Award ID: ES/L008955/1. GW and KET, Award ID: ES/S007113/1.

## AUTHOR CONTRIBUTIONS

PS: Conceptualization: Equal; Data curation: Lead; Formal analysis: Lead; Investigation: Lead; Methodology: Lead; Project administration: Lead; Visualization: Lead; Writing – original draft: Lead; Writing – review & editing: Lead. JF: Investigation: Supporting; Project administration: Supporting; Writing – original draft: Supporting. GW: Funding acquisition: Lead; Methodology: Equal; Supervision: Equal; Writing – review & editing: Equal. EP: Funding acquisition: Equal; Methodology: Equal; Supervision: Equal; Writing – review & editing: Equal. KET: Conceptualization: Equal; Methodology: Equal; Supervision: Lead; Writing – review & editing: Equal.

## Note

^1^ A formal description of the model is outside the scope of the current article; for details see Nagai et al. (2006).

## Supplemental Materials

https://osf.io/fqp8z/

https://osf.io/e73fw
